# Summary of the National Advisory Committee on Immunization (NACI) Rapid Response—Interim guidance on the use of Imvamune in the context of monkeypox outbreaks in Canada

**DOI:** 10.14745/ccdr.v48i78a09

**Published:** 2022-07-07

**Authors:** April Killikelly, Nicholas Brousseau

**Affiliations:** 1 Public Health Agency of Canada, Centre for Immunization and Respiratory Infectious Diseases, Ottawa, ON; 2 Institut national de santé publique du Québec, Direction des risques biologiques, Québec, QC

**Keywords:** National Advisory Committee on Immunization, monkeypox, Canada, Imvamune interim guidance

## Abstract

**Background:**

Monkeypox is endemic in Central and West Africa. Cases in non-endemic countries, including Canada, have been increasing since May 2022. Imvamune^®^, a live, non-replicating smallpox vaccine, was approved by Health Canada for active immunization against smallpox and monkeypox infections and disease in adults determined to be at high risk for exposure. The aim of this interim guidance is to consider the use of Imvamune for post-exposure prophylaxis (PEP) and to summarize the available evidence in support of Imvamune use in this specific current context.

**Methods:**

The National Advisory Committee on Immunization (NACI) High Consequence Infectious Disease Working Group (HCID WG) reviewed data on the current status of the monkeypox outbreak, along with additional evidence included in published scientific literature and from manufacturers, regarding the safety, immunogenicity and protection offered by Imvamune. NACI approved these HCID WG recommendations on June 8, 2022.

**Results:**

In brief, NACI recommends that PEP, using a single dose of the Imvamune vaccine, may be offered to individuals with high-risk exposures to a probable or confirmed case of monkeypox, or within a setting where transmission is happening. After 28 days, if an individual is assessed as having a predictable ongoing risk of exposure, a second dose may be offered. Imvamune may be offered to special populations; including individuals who are immunosuppressed, pregnant, breastfeeding, younger than 18 years of age and/or with atopic dermatitis.

**Conclusion:**

NACI has rapidly developed guidance on the use of Imvamune in Canada in the context of many uncertainties. Recommendations may be revisited as new evidence emerges.

## Introduction

Monkeypox virus is a member of the *Orthopoxvirus* genus, which also includes variola virus (smallpox virus), vaccinia virus, cowpox and other poxviruses. The disease is usually self-limiting and resolves within 14–28 days. Symptoms differ from smallpox and may include fever, headache, back pain, myalgia, asthenia, lymphadenopathy and skin lesions/rash. The duration of communicability for monkeypox virus may be up to 2–4 weeks, based on limited evidence of polymerase chain reaction detection of monkeypox in the upper respiratory tract ((1)). Potential complications of monkeypox include secondary bacterial infections, pneumonia, sepsis, encephalitis and vision loss from corneal inflammation. Monkeypox virus may cause severe disease in young children, individuals who are immunocompromised ((2)), and those who are pregnant. Information about monkeypox in people who are pregnant is sparse, but cases of first trimester miscarriage and stillbirths have been reported ((3)). In the current 2022 multi-country outbreak, monkeypox cases may have an atypical presentation including oral, genital, and/or anal lesions with or without fever, or systemic symptoms.

The 2022 multi-country monkeypox outbreak represents the first incidence of broader community transmission in a number of countries outside of certain regions of Africa. According to open-source information, the Québec cases are mainly men 30–55 years of age who presented to sexually transmitted and blood-borne infection clinics in the Montréal area.

Imvamune^®^ (also called Modified Vaccinia Ankara-Bavarian Nordic [MVA-BN], Jynneos^®^, Imvanex^®^) is a non-replicating, third-generation smallpox vaccine manufactured by Bavarian Nordic. Imvamune was approved on November 5, 2020 by Health Canada for active immunization against smallpox, monkeypox and related *Orthopoxvirus* infections and disease in adults 18 years of age and older determined to be at high risk for exposure ((3)). Imvamune differs from previous generations of smallpox vaccines as it is a non-replicating vaccine virus in humans, meaning that based on preclinical studies, it is not able to produce more copies of itself ((4)). Imvamune is stockpiled within Canada’s National Emergency Strategic Stockpile for the purposes of national security due to its potential efficacy against variola, the virus that causes smallpox.

In the context of the rapidly evolving multi-country monkeypox outbreak, the planned task for this Rapid Response was to consider the use of Imvamune for post-exposure prophylaxis and to summarize the available evidence in support of Imvamune use in this specific current context. Unrelated to the current monkeypox outbreak, the National Advisory Committee on Immunization (NACI) was also asked to consider use of Imvamune in laboratory research settings where replicating orthopoxviruses are studied.

Details can be found in the updated NACI advisory committee statement: *NACI Rapid Response—Interim guidance on the use of Imvamune in the context of monkeypox outbreaks in Canada* ((5)).

## Methods

On May 26 and May 27, 2022, monkeypox data were discussed and reviewed by the NACI High Consequence Infectious Disease working group (HCID WG), along with input from the Public Health Ethics Consultative Group, Canadian Immunization Committee, NACI’s Vaccine Safety Working Group and two lesbian, gay, bisexual, transgender, queer or questioning and two-spirit (LGBTQ2S+) stakeholder groups. The HCID WG reviewed data on the current status of the monkeypox outbreak, along with additional evidence included in published scientific literature and from manufacturers, regarding the safety, immunogenicity and protection offered by Imvamune. NACI approved these HCID WG recommendations on June 8, 2022.

## Results

**Table 1** summarizes the very limited evidence upon which the NACI recommendations were based as well as the unknowns for each recommendation area.

**Table 1:** Knowns and unknowns^a^

Knowns		Unknowns
Pre-exposure prophylaxis		
From early-stage clinical trials, most of the reported AEFIs were mild to moderate and resolved within seven days following vaccination. Some cardiac AEFIs were reported in Imvamune^®^ recipients and none were considered serious. Indirect clinical immunological evidence showed that Imvamune was able to generate immune responses by week two after a first dose, and comparable immune responses to previous generation smallpox vaccines after two doses by week six. Indirect clinical evidence showed that two doses of Imvamune was able to generate protection from symptomatic vaccinia (*Orthopoxvirus* related to monkeypox). Imvamune immune responses may decrease after two years. Immune responses to booster doses of Imvamune were fast and to the level of primary series responses.		The efficacy or effectiveness of Imvamune PrEP against monkeypox infection or disease is unknown. There were no direct efficacy or effectiveness data for Imvamune against monkeypox. There was limited safety data for Imvamune PrEP. The degree to which preclinical, immunological or *Othopoxvirus* data was predictive of Imvamune protection or durability against monkeypox is unknown. The number of doses, or dose interval, for optimal protection by Imvamune PrEP in immunocompetent adults without underlying medical conditions is unknown. The protection offered by previous smallpox vaccination (potentially from decades ago) and the best use of Imvamune PrEP in those previously vaccinated is unknown. Due to the limited context of Imvamune use (i.e. clinical trials), low frequency AEFIs (frequency fewer than one in 10,000) are unknown. The degree to which previous infection or vaccination impacts the efficacy/effectiveness and safety Imvamune PrEP is uknown.
Post-exposure prophylaxis		
Indirect preclinical immunological evidence showed that Imvamune PEP was able to generate comparable immune responses to previous generation smallpox vaccines. Based on historical data for first generation vaccination against variola, the earlier the PEP was given, the better the protection from disease. Indirect clinical immunological evidence from PrEP studies showed that Imvamune was able to generate immune responses by week two after a first dose, and comparable immune responses to previous generation smallpox vaccines after two doses by week six.		The safety, efficacy or effectiveness of Imvamune PEP against monkeypox infection or disease is unknown. There were no direct safety, efficacy or effectiveness data for Imvamune PEP against monkeypox. The number of doses, dose interval or timing from exposure, for optimal protection by Imvamune PEP in immunocompetent adults without underlying medical conditions is unknown. The degree to which previous infection or vaccination impacts the efficacy/effectiveness and safety Imvamune PEP is unknown.
Special populations		
Immunosuppressed individuals	Based on limited clinical study, safety among people living with HIV (CD4 at least 100 cells/µL) and HSCT seemed comparable with non-immunosuppressed controls. Compared to people without HIV, individuals living with HIV may have had lower immune responses to one dose of Imvamune and may have had decreased durability of immune responses. Imvamune was well tolerated in 20 individuals who received hematopoietic stem cell transplant.	The efficacy or effectiveness of Imvamune PEP or PrEP against monkeypox infection or disease is unknown. There were no direct efficacy or effectiveness data for Imvamune in this population against monkeypox. There were limited safety data for Imvamune PrEP. It is not yet clear if some immunocompromised groups will be less protected by the vaccine and may require specific vaccine doses, intervals or antigen levels.
Pregnant and breastfeeding individuals	No safety concerns were identified during limited clinical and preclinical testing of Imvamune.	The safety, efficacy or effectiveness of Imvamune PEP or PrEP against monkeypox infection or disease is unknown. Imvamune has never been tested in this population. There were no direct safety, efficacy or effectiveness data for Imvamune in this population against monkeypox.
Children younger than 18 years	No safety concerns were identified in limited clinical testing of Imvamune-like vaccines in approximately 2,000 children under 18 years of age.	The safety, efficacy or effectiveness of Imvamune PEP or PrEP against monkeypox infection or disease is unknown. Imvamune has never been tested in this population. There are no direct safety, efficacy or effectiveness data for Imvamune in this population against monkeypox. It is not yet clear if some age groups may require specific vaccine doses, intervals or antigen levels or if there is a minimum age for vaccination.
Individuals with AD	In limited clinical testing, Imvamune was well tolerated in individuals with AD, though individuals with AD may experience a higher frequency of local and systemic reactogenicity compared to those without AD.	The safety, efficacy or effectiveness of Imvamune PEP or PrEP against monkeypox infection or disease is unknown. There are no direct efficacy or effectiveness data for Imvamune in this population against monkeypox. There are limited safety data for Imvamune.

Abbreviations: AEFIs, adverse events following immunization; AD, atopic dermatitis; HSCT, hematopoietic stem cell transplantation; PEP, post-exposure prophylaxis; PrEP, pre-exposure prophylaxis

^a^ Summarized from the *Interim guidance on the use of Imvamune in the context of monkeypox outbreaks in Canada* ((5))

## Recommendations

1. NACI recommends that post-exposure prophylaxis (PEP) using a single dose of the Imvamune vaccine may be offered to individuals with high-risk exposures ((6)) to a probable or confirmed case of monkeypox, or within a setting where transmission is happening. The PEP should be offered as soon as possible and within four days of last exposure and can be considered up to 14 days since last exposure. The PEP should not be offered to individuals who are symptomatic and who meet the definition of suspect, probable or confirmed case. After 28 days, if an individual is assessed as having a predictable ongoing risk of exposure, a second dose may be offered. A second dose should not be offered to individuals who are symptomatic and therefore after medical evaluation meet suspect, probable or confirmed monkeypox case definitions. For individuals who had received a live replicating first or second generation smallpox vaccine in the past and who sustain a high-risk exposure to a probable or confirmed case of monkeypox, a single dose of Imvamune PEP may be offered (i.e. as a booster dose). The benefit of protection against infection should be discussed with a healthcare provider and weighed against the potential risk of recurrent myocarditis for individuals with a history of myocarditis/pericarditis linked to a previous dose of live replicating first and second generation smallpox vaccine and/or Imvamune; a precautionary approach is warranted at this time until more information is available.
2. NACI recommends that Imvamune pre-exposure prophylaxis (PrEP) may be offered to personnel working with replicating orthopoxviruses that pose a risk to human health (vaccinia or monkeypox) in laboratory settings and who are at high risk of occupational exposure. If Imvamune is used, two doses should be given at least 28 days apart. A booster dose may be offered after two years if the risk of exposure extends beyond that time. This recommendation does not apply to clinical diagnostic laboratory settings at this time, due to very low risk of transmission. For immunocompetent individuals who have received a live replicating first or second generation smallpox vaccine in the past and who are at high risk for occupational exposure, a single dose of Imvamune may be offered (i.e. as a booster dose), rather than the two dose primary vaccine series. This single Imvamune dose should be given at least two years after the latest live replicating smallpox vaccine dose. In consultation with a physician, the benefit of protection against infection should be weighed against the risk of recurrent myocarditis for individuals with a history of myocarditis/pericarditis linked to a previous dose of live replicating first and second generation smallpox vaccine and/or Imvamune; a precautionary approach is warranted at this time until more information is available.
3. NACI recommends that Imvamune vaccine may be offered to the following populations, if recommended to receive vaccine based on exposure risk: individuals who are immunocompromised due to disease or treatment; individuals who are pregnant; individuals who are lactating; children/youth; and individuals with atopic dermatitis.
4. NACI recommends that Imvamune given as PEP or PrEP should not be delayed due to recent receipt of a messenger ribonucleic acid (mRNA) coronavirus disease 2019 (COVID-19) vaccine. If vaccine timing can be planned (i.e. prior to employment within a research laboratory), NACI recommends that Imvamune be given at least four weeks after or before an mRNA vaccine for COVID-19.

## Conclusion

Based on limited available evidence, NACI and other stakeholders were able to develop and deliver guidance on the use of Imvamune in the context of the monkeypox outbreak in Canada 22 calendar days from the first case reported in Canada. These recommendations were made in the context of many unknowns and uncertainties and may be revisited as new evidence emerges.
